# Registrations of Disease in England

**Published:** 1858-01

**Authors:** 


### THE
### SANITARY REVIEW.

JANUARY 1858.

### THE REGISTRATION OF DISEASE IN ENGLAND.*

WHOEVER will take the trouble attentively to think over the whole subject of epidemic diseases, their origin, their course, their causes, will not fail to be struck with the certainty of the fact that, before any great general law in relation to epidemics can be discovered, they must be observed on a grand scale for some considerable period of time, and in a systematic and scientific manner.

There are essential differences between the observation of an epidemic, and the observation of other natural phenomena, such as the rolling of a planet, the radiation of light, the development of electricity, and the like.

These latter grand phenomena in nature are always present; the phenomena of epidemics are fleeting. If we would unravel the mysteries of either class of these phenomena, we must do so by the agency of a multitude of minds. The process of observation is, however, different in each case. In the investigation of the permanent phenomenon, time is allowed; and one single individual, in one age, may work usefully and progressively, since he may start on the last foot-mark of him who preceded, and may have the problem for solution always before him. In the investigation of the fleeting phenomenon, the single individual becomes a nullity; and the difficulty of discovering its nature can only be overcome by bringing the whole competent mental strength of the time to bear upon it.

M. Jules Guérin, in drawing up a report for the Imperial Academy of Medicine, on the subject of Miliary Fever, added

* The Registrar-General's Returns since 1850.
Weekly Returns of the Health of the Metropolis. By the Medical Officers of Health. Nos. 1 to 34.
Memoirs of the Imperial Academy of Medicine, Paris. GUERIN, On Miliary Fever. Vol. XVII. 1853-4.
Weekly Return Books of Union Medical Officers, collected from various Unions.

some general observations kindred to the above on the study of epidemics.

"The study of epidemics, submitted as yet to too few regulations, and left too much to individual choice, has never rendered the services it is capable of yielding to science and to mankind. Considered in their highest characteristics, epidemics are the great manifestations of a sole and identical cause, which impresses an uniform and well marked character on all its products, leaving to surrounding etiological actions only a limited and secondary influence. It results from this etiological predominance of the great epidemical cause, that all the morbid individualities in the same epidemic have a general and principal resemblance, and exhibit only particular and accessory differences.

"As a consequence of this general proposition, epidemics become immense centres of observation, in which the extent, number, and diversity of the facts, and the variable conditions of their manifestations, are constantly throwing light upon the most obscure pathological problems. That which is only seen imperfectly or in separate letters, in sporadic diseases, may be read in large characters and entire words during epidemic visitations. The cause of the sporadic disease is almost always feeble and isolated; and, dividing its influence with the ordinary etiological conditions of age, sex, constitution, and season, it gives rise to mixed and ill characterised products, the comparison of which is a matter of difficulty. In a word, in sporadic diseases the elements of uniformity are almost equally balanced by those of diversity, and the products are as the factors. In epidemics the essential cause is one and absolute, and of sufficient energy to take the lead. In the presence of these grand etiological manifestations, the mind requires to make scarcely any effort to seize at once, and to compass in its entirety, that which observation in ordinary diseases is obliged to seek from its useful auxiliaries, analysis, comparison, and numeration.

"Epidemics are in some sort generalisations already prepared; while, in the case of sporadic diseases, induction only obtains the object of generalisation with great labour, by aid of a series of observations made in all times and in all places. But, in order that all possible benefit may be derived from these great morbid revelations, and that we may not lose ourselves in the labyrinth of this mine of inexhaustible riches and fertility, it is necessary that science should have her routes traced out, and that the travellers passing along these should possess a watchword, and be able to harmonise their efforts toward definite ends, if not toward the same ends, so that every acquired idea may form the point of departure for that which remains to be acquired. But, instead of a system of observation based on these principles, what do we see? The greatest divergence in views, efforts, and means! Every one proceeding on just as he lists, generally quite by chance, and devoid of light or object: and such uncertainty and divergence at the point of departure has only for its effect a perpetuation of the uncertainty, if not a complete contradiction in the results.

"The first thing to do", continues this eloquent writer, "before commencing the study of an epidemic, should be to inquire what is already known concerning the disease. Has it already shown itself before, and in the same localities? Does it exhibit the same form and characters, and call for the same treatment? Have observation and experience confirmed or modified conclusions already acquired? Have they revealed new particulars, either for the better definition or better treatment of the disease? This, according to our view, would be the better means of attaching the present to the past, and of continuously fertilising the acquisitions of prior knowledge and observation by means of the present. And let it be understood that we have not in view mere bibliographical learning, the so frequently sterile researches of erudition; but the real product of observation and experience, attached to each other through time aad space, the links of the same chain, constituting what one would willingly call the historical formula of an epidemic."

Such is the grand scheme for the collection of facts relating to epidemics suggested by M. Guèrin. To follow out the suggestion to its ultimate consequences, certain fixed rules for research should be universal.

The first point in the observation of epidemics is unity of purpose and of design. Observers should be made aware what are the chief objects of study in relation to epidemics, and how and when these objects are to be carried out. Let us glance at these important questions.

The study of an epidemic admits of being pursued at two different times: 1. When the epidemic itself is absent; 2. When it is present. The process of observation will necessarily vary very much in each case; and this is the first thing to be remembered in the organisation of a correct system of observation. We believe, indeed, that half the want of success that has occurred in the study of epidemics, has arisen from the fact that these diseases are only thought worthy of consideration, by the majority of observers, at such times as they are present. Hence we see during a serious epidemic visitation all thoughts roused, and every eye working; but no sooner has the visitation fled by, than, as if wearied outright by the duties of the task, all thought rests, and every eye sleeps.

We need not say that this is not a faithful, fair, or calm mode of conducting a philosophical inquiry. The epidemiologist is evidently behind the astronomer in the matter of carrying on scientific investigations. If a comet suddenly comes flaming out of space, and, doubling the sun, hastens back again into the invisible, the world looks on with amazement and terror. But the astronomer, unmoved as ever, merely turns his telescope from planet, asteroid, or nebulæ, to the new visitor, watches him through his course with eager attention, notes down his wanderings, and, when he is quite lost to view and to the world, begins for the first time to calculate his nature, his wherefrom, and his return. We have often thought that if the eclipses of the sun or moon had always been observed in the same manner as physicians observe transient diseases, the nature of an eclipse would have remained as yet an unsolved problem. Taken simply as a natural phenomenon, the eclipse resembles an epidemic in many particulars; it comes suddenly; it comes on the vulgar irregularly; it lasts only for a limited period; it rouses the fears of the common mind to the fullest extent; and, amongst those who do not understand the subject in its simple true form, every kind of vague and absurd theory is set up in reference to its causes. It is obvious, however, to the learned, that the mere observance of an eclipse *per se* could never have explained the reason of its appearance, and have proved the truth of that reasoning, by the prediction of a recurrence of the same fact. More was required. The facts of the phenomenon had to be taken at the time; the reason of the phenomenon had to be collected from more general observations and relationships, from after study, and from a comprehension of the order and plan of the stellar universe as a whole.

In instituting this comparison between an astronomical and an epidemiological inquiry, we do not wish to lay down an absolute parallelism. We know, indeed, that such does not exist. But there is an analogy in the two studies; and our object is to show that the time in which an epidemic is present is not the only time when it admits of being studied, and that the discovery of the laws of an epidemic visitation can be attempted only when the epidemic is fairly past, and when the facts which it has presented are fully laid out before the reasoner.

It follows, then, that one chief object of study in relation to an epidemic that has recently disappeared, is the consideration, arrangement, and analysis of the facts which such an epidemic has presented—the construction, as M. Guérin would say, "of the historical formula of the disease."

Together with these noted facts, another necessary inquiry in regard to epidemics, and which admits of being followed during their absence, consists in taking a broad physiological view of the great laws by which health is maintained, and in comparing these normal processes with those modified conditions observable in the subject suffering from the epidemic disorder.

From the consideration of those great and philosophical studies which should be carried out in the absence of an epidemic, and which are suitable, and indeed possible, to but a limited number of minds, we turn now to the less poetical, more matter-of-fact part of this subject—the mode of conducting observations during the existence of a spreading disorder.

In this labour four elements are required.

1. The whole competent mental strength of the district or country in which the epidemic exists.

2. Uniformity in the system of observation.

3. A ready and easy mode of recording observations in a simple and scientific manner.

4. A properly constituted plan, by which the facts observed may be at once collected and submitted to analysis and publication.

These objects, the accomplishment of which would do so much for science and for the world, have long been aimed at, but never satisfactorily achieved. The nearest approach to such a system has perhaps been attempted in France with most success. The plan pursued in France is as follows: Certain medical officers are distributed over the different departments, and are called physicians for epidemics. Upon the prefect of a department receiving information from any of the mayors that an epidemic prevails in his locality, he instructs one of these physicians to repair to the spot, to investigate the causes of the outbreak, to superintend the application of remedial agencies, and to report the result of his observations. In this way a large body of valuable facts is accumulated by competent observers; but, as some of the local practitioners feel considerable jealousy at this interference, and as in some of the poorest districts there are no practitioners at all, it follows sometimes that the authorities are not informed of the existence of an epidemic until it has already long prevailed, or has even passed away, so that few correct data can be obtained respecting it. All reports are forwarded by the physicians for epidemics to the Minister of Agriculture and Commerce.

In Germany, the same has been tried on a smaller and private scale; but the results are, as far as we can learn, almost *nil*. In England, until within the last few years, there was absolutely no system whatever that could be turned to a proper and correct account, in relation to epidemics, until the institution of the mortality returns of the Registrar-General. These returns are invaluable, as showing in some degree, and on a grand scale, the mortality of epidemics, their prevalence, their course, their effects on the sexes, their relationship to season, and it may be, in an imperfect manner, their connexion with meteorological conditions. Still, these returns are only absolutely valuable as regards mortality; this, their original object, is their only direct good. They exhibit accurately *the balance paid over by disease to death, but not that which is paid over by health to disease.*

In relation to special epidemics, attempts have been made by the Epidemiological Society to collect facts from a large number of observers. These have been in some degree successful; but not sufficiently so to give rise to any great "historical formula". The same may be said of such endeavours as have been made by the General Board of Health.

In the *Association Medical Journal*, a special inquiry was commenced in 1853, and continued for some time with great labour and intelligence. The attempt here was to show the connexion that exists between certain meteorological phenomena and the progress of disease. There can be no doubt as to the great merit of these tables; but, to have made them really useful, the staff of observers should have been increased a hundred fold, and the basis of inquiry should have been wider.

When this REVIEW commenced, now three years ago, it took as one of its leading objects the collection of the histories of epidemics from various observers. Commencing with some dozen learned coadjutors, the Editor found the system generally approved; and now the staff of observers is not less than forty in number. Of the ultimate value of these researches there can be no doubt.

The Association of Medical Officers of Health have more recently instituted a weekly Register of the Diseases in the Metropolis, and are doing excellent service.

Thus we have in England, at the present time, three registers of epidemics: the Registrar-General's Returns; the Local Epidemic Returns of this REVIEW; and the Returns of the Medical Officers of Health in London.

The first is strictly a mortality register; the two latter are right in principle, but deficient in extent.

To supply this latter deficiency, the exertions of a *few* men are insufficient; a grand scheme is required: and our object in this article is to show that in this country the finest possible organisation for the observation of epidemics at this moment exists, and waits only for the word of command and the judicious counsel of a learned committee to become a great and living system.

The plan we have to propose is nothing more than that of making every parochial medical officer's weekly return book the accurate official record of the epidemic and other diseases occurring in his district.

Let us for a moment glance at the extent and capabilities of this proposed plan.

In the first place, the whole of England is fairly divided amongst the union medical officers. The poor of the districts are mainly under their care; and the inference, therefore, is tolerably safe, that no important epidemic could occur in any part without being duly noticed.

Secondly, the points of observation are numerous. We find, from a return made by Mr. Baines to the House of Commons in the year 1853, that there are not less than 3,233 parochial medical officers in England and Wales.

Thirdly, the observers thus occupied are of all men the most fitted to the task. They are stationed in their special localities for great lengths of time; they are well acquainted with the characters of the localities, with the manners, habits, and diseases of the people; and they are, as a general rule, amongst the most zealous, humane, and educated members of the community.

Fourthly, and this is the most important of all, the parochial medical officer, as we know from experience, could return his observations with the least possible labour. He does, in fact, already make this return, in great part, without any reference to its more important bearings. In order to supply his Board of Guardians with correct information on the condition of the poor under his charge, he is furnished with a weekly return sheet. In this sheet it is his duty to enter the name, the sex, the age, the residence, and the disease of each of his poor patients. He has, further, to state the days on which he visited the patient, the diet required, and such other general observations as he may think proper.

Now, having ourselves made use of these return books in collecting facts on disease, we can speak with certainty as to their value, even in their present rudely constructed form; and could these records, as they now exist, be brought together, we might glean from them a large amount of valuable information. But if these 3,233 weekly records, thus rudely drawn up, and for trifling objects, comparatively speaking; if these records were so modified as to become really scientific registers of epidemic diseases; if, thus modified, they were regularly transmitted to some central authority, who could use them for a definite purpose, what truly important tablets of disease they would become, what firm bases for the historical formula! There would then be no disease registration in the world like that of England.

As regards the forms themselves, it is worthy of remark that, in their present state, they perform only a temporary purpose. They supply the guardians of unions with information for the week, and are then forgotten. We believe that, under any new system, which should require them to be sent away after the meeting of the board, a duplicate need scarcely be retained.

The modifications that would be required in the returns themselves are few and simple. In the first place, in every return book there should be drawn on the fly-leaf a map of the observer's district, and a brief description of the geological character, extent, latitude, longitude, produce, number of towns, population, and the like. These particulars, once drawn out and printed, would remain for many years without any material alteration. At all events, they would give the medical observer no trouble, though rendering his reports infinitely valuable.

The modifications required in the weekly returns are very few. The columns would remain unaltered. In the columns, however, now set apart merely to express the number of visits paid, an additional letter or two should be introduced, to indicate certain important facts. The V or the X might still stand to represent the visit; but the letters D D should be inserted, to show the day of commencement of disease; the single D to represent the day of death; the letter C to show when convalescence commenced; and R to point out the time of complete recovery.

By an extension of the day columns, and by a few cross lines, an arrangement might be formed for the recording of the meteorological readings of the week; and the date at the top, as given in present returns, would decide the question of seasons. The two last columns might well remain unchanged; the one for necessaries would show the diet of the patient; the one for observations would leave room for notes on treatment, hygienic conditions, or other special points. This column would be better if left rather larger than it now stands in the return book.

Such modifications as are required are shewn briefly in the accompanying table.

For the mere observance of epidemics, then, there exists in this country every convenience ready to hand, and laid out on a plan magnificently large and comprehensive. The successful execution of the scheme depends on the will and exertions of the Government, by whom alone it can be made available.

*First.* The medical officer, on taking charge of any district, should receive the drawing up of a correct disease return as an imperative part of his duties, for the performance of which he should have just and fair remuneration.

### RETURN AS IT AT PRESENT STANDS.

Form P. *Week ending Friday*, 13*th day of November* 1857.

| Name. | Age. | Residence. | Parish to which chargeable. | Nature of Disease. | Days when attended, or when Medicines were furnished. | | | | | | | Necessaries ordered to be given to the Patient. | Present state or termination of the case. | Observations. |
|---|---|---|---|---|---|---|---|---|---|---|---|---|---|---|
| | | | | | Sat. | Sun. | Mon. | Tu. | Wed. | Th. | Fr. | | | |
| John Williams | 10 | Chiltern | Chiltern | Scarlet fever | x | x | .. | x | x | x | .. | 2 oz. wine daily | Serious | First case imported. |
| **REMODELED FORM.** | | | | | | | | | | | | | | |
| Charles Jacob | 12 | Chiltern | Chiltern | Scarlet fever | V, DD | V | V | V | V | V | D | 2 oz. wine daily | .... | Disease of malignant type. This was the second case, conveyed clearly by infection. |
| John Jones - | 24 | St. George | St. George | Rheumatism | V | V | .. | V | .. | V, C | .. | .... | Convalescent | A mild case. |
| W. Thompson | 16 | Dunwich | Dunwich | Measles | V, DD | V | V | V | V | V | V | .... | Improving | Bronchial symptoms severe. The epidemic has been prevalent during last 2 months. |
| Mary Atkins - | 36 | Dunwich | Dunwich | Measles | .. | V, DD | V | .. | V | .. | V | 1 oz. wine daily | Nearly well | A mild case. |
| Thos. Williams | 14 | Stone | Stone | Scarlet fever | .. | .. | V, DD | V | V | V | V | 2 oz. wine daily | Serious | The epidemic spreading rapidly. |
| Direction of wind .............................. | | | | | NE. | N. | NW. | N. | N. | N. | NE. | | | |
| Ozone ........................................ | | | | | 1 | 0 | 1 | 3 | 0 | 1 | 5 | | | |
| Mean temperature ............................ | | | | | 47° | 49° | 50° | 40° | 41° | 43° | 46° | | | |
| Barometer .................................... | | | | | 29·7 | 29· | 29·6 | 30· | 29·8 | 29·7 | 29· | | | |
| Rain in twenty-four hours (inches) ............ | | | | | ·004 | ·012 | ·006 | ·010 | ·007 | ·000 | ·002 | | | |

*Secondly.* The weekly return, immediately after it has served its local purpose, should be dispatched at once to London, to be submitted to a Registrar-General of Disease, and to be reported upon weekly after the manner of the reports of births and deaths, and the Registers of the Medical Officers of Health.

With these remarks, we leave the suggestions contained in this paper in the hands of the reader. Pile upon pile, mouldering away amongst dust and rubbish in the cellars of workhouses, and in the surgeries of medical men, lie what might become the greatest records of disease ever published—the veritable histories which Government has sporadically sought after at great pains, great cost, and little advantage. Sometimes these documents are sold as waste paper; and, instead of fulfilling their legitimate and useful objects, are turned to the clothing of perishable butter and briny bacon. Surely this veritable outspreading of pearls before dead swine ought not to continue.

We feel it possible that one objection may be made to these records. It may be said that they will not represent the epidemics of a whole population. To this we answer, that the number of cases occurring in any given epidemic is of no importance whatever, if the course of the disease be observed over an immense and isolated tract of land, such as England; if its general characters be carefully described; and if the conditions which peculiarly favour its manifestation be accurately pointed out. From three thousand weekly returns, all constructed on the same principles, these and many other facts could not fail to be elicited; while from the facts themselves, carefully stored up for some years, might eventually be wrought out the historical formulæ of not one, but many, of the epidemic diseases.

